# Systemic Administration of *Calea pinnatifida* Inhibits Inflammation Induced by Carrageenan in a Murine Model of Pulmonary Neutrophilia

**DOI:** 10.1155/2020/4620251

**Published:** 2020-01-25

**Authors:** Bruno Matheus de Campos Facchin, Julia Salvan da Rosa, Ana Beatriz Gobbo Luz, Yeo Jim Kinoshita Moon, Tamires Cardoso de Lima, Rosana Casoti, Maique Weber Biavatti, Eduardo Monguilhott Dalmarco, Tânia Silvia Fröde

**Affiliations:** ^1^Department of Clinical Analysis, Center of Health Sciences, Federal University of Santa Catarina, Campus Universitário, Trindade, Florianópolis, 88040-970 SC-, Brazil; ^2^Department of Pharmaceutical Sciences, Center of Health Sciences, Federal University of Santa Catarina, Campus Universitário, Trindade, Florianópolis, 88040-970 SC-, Brazil; ^3^Department of Pharmaceutical Sciences, University of São Paulo, Ribeirão Preto, SP-, Brazil

## Abstract

**Objective:**

The aim of this study was to investigate the anti-inflammatory effects of the crude extract (CE), derived fraction, and isolated compounds from *Calea pinnatifida* leaves in a mouse model of pulmonary neutrophilia.

**Methods:**

The CE and derived fractions, hexane, ethyl acetate, and methanol, were obtained from *C*. *pinnatifida* leaves. The compounds 3,5- and 4,5-di-*O*-*E*-caffeoylquinic acids were isolated from the EtOAc fraction using chromatography and were identified using infrared spectroscopic data and nuclear magnetic resonance (^1^H and ^13^C NMR). Leukocytes count, protein concentration of the exudate, myeloperoxidase (MPO) and adenosine deaminase (ADA), and nitrate/nitrite (NO_*x*_), tumor necrosis factor-alpha (TNF-*α*), interleukin-1-beta (IL-1*β*), and interleukin-17A (IL-17A) levels were determined in the pleural fluid leakage after 4 h of pleurisy induction. We also analyzed the effects of isolated compounds on the phosphorylation of both p65 and p38 in the lung tissue.

**Results:**

The CE, its fractions, and isolated compounds inhibited leukocyte activation, protein concentration of the exudate, and MPO, ADA, NO_*x*_, TNF-*α*, IL-1*β*, and IL-17A levels. 3,5- and 4,5-di-*O*-*E*-caffeoylquinic acids also inhibited phosphorylation of both p65 and p38 (*P* < 0.05).

**Conclusion:**

This study demonstrated that *C*. *pinnatifida* presents important anti-inflammatory properties by inhibiting activated leukocytes and protein concentration of the exudate. These effects were related to the inhibition of proinflammatory mediators. The dicaffeoylquinic acids may be partially responsible for these anti-inflammatory properties through the inhibition of nuclear transcription factor kappa B and mitogen-activated protein kinase pathways.

## 1. Introduction

A variety of global diseases are related to nonresolving inflammation. Nonresolution of inflammatory processes is the main cause of many chronic inflammatory diseases, such as atherosclerosis, inflammatory bowel disease, rheumatoid arthritis, chronic obstructive pulmonary disease, and asthma [1]. Asthma is a chronic inflammatory disease characterized by airway hyperresponsiveness and reversible airway obstruction [2]. The mechanisms of eosinophilic inflammation in asthma have extensively been studied; however, little is known regarding noneosinophilic asthma and its development [3]. Recent studies have shown that approximately half of all asthma cases do not exhibit any evidence of eosinophilic inflammation and that some of these individuals present airway neutrophilia [4]. Airway neutrophilia is directly related to the severity of asthma and the refractory treatment for corticosteroids. However, the underlying mechanisms of airway neutrophilia are not well understood but appear to involve the activation and continuous flow of neutrophils [5].

A simple and reproducible experimental model is a basic requirement to evaluate the anti-inflammatory properties of plants used in traditional medicine. Carrageenan- (Cg-) induced inflammation is a commonly used model of local acute inflammation to evaluate the anti-inflammatory action of herbs and to assess the participation of cells and mediators in the inflammatory process. Also, Cg promotes a leukocyte influx due to neutrophils into the mouse pleural cavity 4 h after pleurisy induction [6]. This response is useful to study neutrophilia airway inflammation diseases such as asthma.

Therefore, the search for new therapeutics and bioactive compounds that may be effective in treating patients who do not respond to conventional asthma therapies is necessary. Research based on ethnopharmacological studies can assist in the selection of species with potential biological activities, thus contributing to new drug discoveries [7]. The Brazilian flora presents rich biodiversity, comprising >50,000 plant species, thus becoming an important source of medicinal plants and bioactive compounds. Moreover, the interest in medicinal plant properties has extensively been studied by Brazilian researchers because of the traditional use of medicinal plants in folk Brazilian medicine for various diseases, including inflammatory diseases [8–10].

The genus *Calea* belongs to the Asteraceae family and contains approximately 125 species distributed in tropical and subtropical areas worldwide, with the greatest number of species recorded in Brazil [11]. Studies of *Calea* species demonstrate important pharmacological properties, such as antihypertensive, vasodilator, antiparasitic, hypoglycemic, and anti-inflammatory effects [12–17]. *Calea pinnatifida*, popularly known as “aruca” and “cipó-cruz,” is a perennial and subshrub plant found in Southern Brazil, and has been used in folk medicine to treat giardiasis and amoebiasis [18]. Although this species was not extensively studied thus far, recent studies have shown potential antiproliferative [19] and leishmanicidal effects [20]. The phytochemical profile of *C. pinnatifida* has revealed the presence of fatty esters, sterols, monoterpenes, sesquiterpene lactones, germacranolides, and phenolic acids [21, 22].

Regarding this last class of compounds, the derived caffeoylquinic acids appear to have important anti-inflammatory actions and inhibit the nuclear transcription factor kappa B (NF-*κ*B) pathway, an important intracellular signaling route responsible for the expression of proinflammatory genes involved in the development and progression of many inflammatory diseases, including asthma [23–26]. The anti-inflammatory activity of a mixture containing dicaffeoylquinic acids (diCQAs) has been tested in our research group, which have also demonstrated inhibitory effects on p65 NF-*κ*B phosphorylation [27].

Considering the use of the *Calea* genre in inflammatory diseases and as treatment in folk medicine and the absence of studies confirming these biological effects, we aimed to study the anti-inflammatory actions of *C*. *pinnatifida* and elucidate its possible anti-inflammatory mechanisms using a murine model of pulmonary neutrophilia. Thus, we evaluated the effects of *C*. *pinnatifida* crude extract (CE), derived fractions, and isolated compounds (3,5-di-*O*-*E*-caffeoylquinic acid (3,5-diCQA), 4,5-di-*O*-*E*-caffeoylquinic acid (4,5-diCQA), and 3,4-di-*O*-*E*-caffeoylquinic acid (3,4-diCQA)) on leukocyte migration, protein concentration of the exudate, myeloperoxidase (MPO) and adenosine deaminase (ADA) activities, and nitric oxide metabolites (NO_*x*_), tumor necrosis factor-alpha (TNF-*α*), interleukin-1-beta (IL-1*β*), and interleukin-17A (IL-17A) levels. We also evaluated the ability of isolated *C*. *pinnatifida* compounds on the inhibition of important inflammatory pathways signal via p65 NF-*κ*B and p38 mitogen-activated protein kinases (MAPK) phosphorylation.

## 2. Materials and Methods

### 2.1. Collection and Extraction of Plant Material and Isolation and Identification of the Chemical Constituents

The methods used for the collection and extraction of plant material as well as for the isolation and identification of the chemical constituents were performed as previously described by Lima et al. [28]. *C*. *pinnatifida* leaves were collected in June 2013 from Costa da Lagoa, Florianópolis, Brazil. Botanical identification was provided by Dr. John F. Pruski, New York Botanical Garden, and a voucher specimen has been maintained at the Missouri Botanical Garden Herbarium, St. Louis, Missouri, USA (No: MO-2383318). Fresh leaves of *C*. *pinnatifida* (2.6 kg) were subjected to exhaustive extraction by maceration with 92% ethanol for 15 days at approximately 25°C. The solvent was removed under reduced pressure on a rotatory evaporator yielding 142.0 g of CE. The CE was suspended in H_2_O and partitioned using *n*-hexane (*n*-Hex), followed by dichloromethane (DCM) and ethyl acetate (EtOAc), resulting in three fractions: (1) Hex, 68.5 g; (2) DCM, 7.6 g; and (3) EtOAc, 5.9 g. The remaining aqueous fraction (60.0 g) was subjected to column chromatography (CC) using Amberlite XAD-4 resin as the stationary phase and methanol (MeOH) as the mobile phase, resulting in a MeOH fraction (28.3 g). Subsequently, an aliquot of EtOAc fraction (4.9 g) was further fractionated using vacuum liquid chromatography (VLC) on silica gel and eluted with CH_2_Cl_2_, EtOAc, and MeOH in mixtures of increasing polarity to yield eight subfractions, (A–H). Subfraction C (126.5 mg) was purified using medium-pressure liquid chromatography on a reverse-phase (RP-18) column using a gradient solvent system of H_2_O–MeOH (95 : 5 → 0 : 100) as mobile phase to yield 73 fractions. Fractions F50–53 eluted with H_2_O–MeOH (60 : 40) provided 22.7 mg of compound 2. Finally, an 800.0 mg aliquot of subfraction E was chromatographed over a Sephadex LH-20 column using acetone–MeOH (1 : 1) as a solvent to obtain 37 fractions. Based on the thin layer chromatography (TLC) profile, collected fractions were combined, and an aliquot of the fractions F16–19 (60 mg) was further purified using preparative high-performance liquid chromatography (HPLC) to yield 6.4 and 10.3 mg of compounds 1 and 3, respectively. Chromatography was performed with isocratic elution, a flow rate of 1.5 mL/min, injection volume of 1000 *μ*L, and ACN–H_2_O as mobile phase (20 : 80, *v*/*v*), containing 1% formic acid; ultraviolet (UV) detection was at 325 nm.

### 2.2. Ultra-High-Pressure Liquid Chromatography–DAD–High-Resolution Electrospray Ionization Mass Spectrometry Conditions

Ultra-high-pressure liquid chromatography (UHPLC) analyses were performed using a C18 reverse-phase column ACE® (3 *μ*m particle size, 150 × 3 mm) at 30°C with gradient elution at a flow rate of 0.4 mL/min. The injection volume was 5 *μ*L at concentrations of 0.5 mg/mL of extract/organic fractions in 70% EtOH, and the analysis time was 30 min. The mobile phase eluents consisted of solvents A (H_2_O) and B (acetonitrile (ACN)), both containing 0.1% formic acid. The mobile phase was programmed in a linear gradient as follows: 0–17 min, 2%–55% B; 17–20 min, 55%–100% B; 24–27 min, 100%–2% B; and 27–30 min, 2% B. UV detection was conducted at 214, 254, and 325 nm.

Mass spectra were concurrently acquired in both positive and negative modes, with a mass range of 150–1200 m*/z*. Several parameters were used for all analyses: fragmentation HCD gas off; resolution, 70,000; maximum injection time, 200 ms; capillary temperature, 300°C; AGC target, 3e6; microscans, 1 s; and spray voltage, 3.6 kV.

### 2.3. General Experimental Procedures

Proton nuclear magnetic resonance (^1^H NMR) spectra and correlation maps (HSQC and HMBC) were recorded using a Bruker Ascend 600 spectrometer, operating at 600 MHz for ^1^H and 150 MHz for ^13^C. NMR experiments were conducted in MeOH-*d*_4_ containing a few drops of TMS (0.00 ppm, internal standard), and the data were analyzed utilizing the ACD/labs NMR software. Chemical shifts (*δ*) and coupling constants (*J*) were expressed in ppm and in hertz (Hz), respectively. High-resolution electrospray ionization mass spectrometry (HRESIMS) data were measured on an Accela™ UHPLC equipped with an UV-DAD detector and an Exactive™ Plus Orbitrap mass spectrometer (Thermo Fisher Scientific) using an electrospray ionization source. Data were acquired and processed using the Xcalibur™ software (Thermo Fisher Scientific). Preparative HPLC analyses were performed using a Shimadzu HPLC system (Kyoto, Japan) consisting of a SCL-10ADVP system controller, two LC-10 AD pumps, a SPD-10AV UV detector, and a manual injection system. HPLC analyses were conducted on a C18 reverse-phase column Luna type (10 *μ*m, 250 × 10 mm, Phenomenex®). Silica gel 60 F_254_ (0.04–0.63 *μ*m, 240–400 mesh) utilized for VLC, and CC was acquired from Vetec, Brazil; analytical TLC was obtained from Silicycle; Sephadex LH-20 was purchased from Tedia Brazil; and Amberlite XAD-4 was acquired from Supelco (Sigma Aldrich, Pte. Ltd., Singapore). HPLC grade ACN was acquired from Dist (Florianópolis, SC, Brazil).

### 2.4. Animals

For the experiments, female Swiss mice, weighing between 18 and 22 g, were used. The animals received food and water *ad libitum* and were housed under controlled light (12 h light/dark cycle) and temperature (20 ± 2°C). All procedures performed in this study were in accordance with the ethical standards of the institution or practice in which the studies were conducted. In this study minimum number of animals were used to be consistent the statistical analysis. This protocol was approved by the ethics committee for Animal Research at Federal University of Santa Catarina (CEUA-Protocol PP00757/CEUA/2012).

### 2.5. Carrageenan-Induced Pleurisy

Pleurisy was induced by an injection of 0.1 mL carrageenan (Cg, 1%) according to previously described methods [6]. After 4 h, the mice were sacrificed, and the pleural cavity was exposed and washed with 1.0 mL heparinized (20 UI/mL) phosphate-buffered saline (pH 7.6). The pleural samples from different groups of mice were used to quantify leukocytes, protein concentration of the exudate, MPO and ADA activities, NO_*x*_ levels, and cytokine levels (TNF-*α*, IL-17A, and IL-1*β*). The lung tissues were collected to evaluate the effects of the isolated compounds on p65 NF-*κ*B and p38 MAPK phosphorylation.

### 2.6. Experimental Protocol

For dosages used in animals, the analysis of the dose-response curve was performed. In this protocol, different groups of mice were treated with different doses of CE (25–100 mg/kg), derived fractions (Hex, 5–25 mg/kg; MeOH, 5–25 mg/kg; and EtOAc, 2.5–25 mg/kg), and isolated compounds (3,5-diCQA, 1–5 mg/kg; 4,5-diCQA, 1–5 mg/kg; and 3,4-diCQA, 1–5 mg/kg) administered via the intraperitoneal route (i.p.) 0.5 h before pleurisy induction. The inflammatory parameters, exudate protein concentration, and leukocyte activation were evaluated after 4 h.

To evaluate the time course profiles, the lower dose of CE that caused the most significant inhibition of leukocyte activation and protein concentration of the exudate was selected. In this protocol, different groups of animals were treated with 50 mg/kg of CE 0.5, 1, 2, and 4 h before Cg injection. Inflammatory parameters were analyzed 4 h after pleurisy induction. The time course profile of CE (50 mg/kg) indicated that the anti-inflammatory activity on leukocyte activation and exudate protein concentration occurred when CE was administered 0.5 h before the pleurisy induction (results not shown). The results of the time course profiles obtained with CE were extended to the fractions and isolated compounds.

Based on the results obtained in the abovementioned protocols, we selected the single doses of CE (50 mg/kg), fractions (Hex, 10 mg/kg; MeOH, 10 mg/kg; and EtOAc, 5 mg/kg), and isolated compounds (3,5-diCQA, 2.5 mg/kg and 4,5-diCQA, 5 mg/kg); these were all administered 0.5 h before Cg-induced pleurisy and were used to evaluate other inflammatory parameters. The inflammatory parameters were also analyzed 4 h after pleurisy induction.

A group of animals was concomitantly challenged only with Cg (1%) and was considered the positive control group, whereas the negative control group received only sterile saline (0.95% NaCl); both groups were administered via i.p. Dexamethasone (Dex, 0.5 mg/kg, i.p.) and indomethacin (Indo, 5 mg/kg, i.p.) were administered in different groups of animals as anti-inflammatory reference drugs in all experimental procedures.

### 2.7. Quantification of Leukocytes and Exudate Protein Concentration

After sacrificing the animals with pentobarbital overdose (180 mg/kg), fluid leakage from the mouse pleural cavity was collected for measuring the total and differential leukocytes count and protein concentration of the exudate. The total leukocyte count was performed using a veterinarian automatic counter (MINDRAY, BC-2800 Vet, Nanshan, Shenzhen, China). For differential leukocytes count, 50 *μ*L aliquots were centrifuged in cytospin (Cytopro® Cytocentrifuge Wescor, Model: 7620 USA) and stained using the May Grunwald–Giemsa method. The differential cell count was determined using an optical microscope with an oil immersion objective (1000×). The results are expressed as relative fold change in relation to saline group.

The exudate protein concentration was measured by amount of Evans blue present in the fluid leakage from the mouse pleural cavity. In this protocol, a group of animals received an intravenous injection of Evans blue solution (25 mg/kg) 0.5 h before the induction of pleurisy. The exudate protein concentration were determined by interpolation from a standard curve of Evans blue (0.01–50 *μ*g/mL) by colorimetric measurements (620 nm) in an enzyme-linked immunosorbent assay (ELISA) plate reader (Organon Teknika, Roseland, NJ, USA). The results are expressed in *μ*g/mL.

### 2.8. Quantification of MPO and ADA Levels

The analysis of MPO and ADA levels were performed according to previously described methods [29, 30].

For the determination of MPO activity, 100 *μ*L samples of fluid leakage from the mouse pleural cavity were treated with hexadecyltrimethylammonium bromide (0.5%), and this mixture was frozen and thawed for three cycles. Subsequently, the samples were centrifuged at 40,000 g for 15 min at 4°C. In total, 20 *μ*L of each of the sample supernatants were added to 180 *μ*L of the reagent solution (0.167 mg/mL o-dianisidine·2HCl and 0.0005% H_2_O_2_), transferred to the enzyme immunoassay plates, and incubated at 37°C for 15 min. The enzymatic reaction was stopped by adding 15 *μ*L sodium azide (1%). Following a 10 min incubation at 37°C, the enzymatic activity was determined by interpolation from a standard MPO curve (0.7–140 mU/mL) by colorimetric measurements (450 nm) in an ELISA plate reader (Organon Teknika, Roseland, NJ, USA). The results are expressed in mU/mL.

For quantification of ADA activity, fluid leakage samples from the mouse pleural cavity containing the enzyme were transferred to test tubes, and the reaction was started by adding 250 *μ*L of a buffered adenosine solution (21 nM adenosine, 35 mM NaH_2_PO_4_·H_2_O:15 mM Na_2_HPO_4_·12H_2_O, pH 6.5). After incubation for 60 min at 37°C, 500 *μ*L of a phenol/sodium nitroprusside solution (106 nM C_6_H_5_OH; 0.17 nM C_5_FeN_5_Na_2_O·2H_2_O) and 500 *μ*L of an alkaline sodium hypochlorite solution (11 nM NaOCl; 125 nM NaOH) were added to each sample and incubated at 37°C for 30 min. Subsequently, the enzymatic activity was determined by interpolation from a standard ADA curve (10–50 U/L) by colorimetric measurements (630 nm) in an ELISA plate reader (Organon Teknika, Roseland, NJ, USA). The results are expressed in U/L.

### 2.9. Quantification of NO Metabolites (NO_*x*_) Levels

Quantification of the NO products in the fluid leakage from mouse pleural cavities was performed according to previously described methods [31].

Fluid samples (100 *μ*L) were transferred to cuvettes, and 0.05 M vanadium chloride (150 *μ*L) in 1.0 M HCl (50 *μ*L) was added to reduce nitrate to nitrite. Griess reagent (300 *μ*L; 0.004 M naphthyl ethylenediamine dihydrochloride in H_2_O and 0.06 M sulfanilamide in 0.03 M H_3_PO_4_, 1 : 1 *v*/*v*) was then immediately added. After incubation at 37°C for 45 min, the reaction was transferred to an ELISA microplate. Nitrite levels were estimated by interpolation from a standard curve of sodium nitrite (0–150 *μ*M) by colorimetric measurements (540 nm) in an ELISA plate reader (Organon Tecknika, Roseland, NJ, USA). The results are expressed in *μ*M.

### 2.10. Quantification of TNF-*α*, IL-17A, and IL-1*β* Levels

TNF-*α*, IL-1*β*, and IL-17A levels in the fluid leakage from the mouse pleural cavity were determined using commercial kits containing monoclonal antibodies for each cytokine. The analyses were performed using ELISA, according to the manufacturer's instructions (TNF-*α*, eBioscience, Inc., San Diego, CA, USA, Cat 88-7342-29; IL-17A, eBioscience, Inc., San Diego, CA, USA, Cat 88-7971-29; IL-1*β*, eBioscience, Inc., San Diego CA, USA, Cat 88-7913-29). The intra- and interassay coefficients of variation (CV) for TNF-*α*, IL-1*β*, and IL-17A were as follows: (1) intra-CV for TNF‐*α* = 7.87 ± 0.9%, IL‐1*β* = 6.27 ± 0.4%, and IL‐17A = 7.57 ± 1.7% and (2) inter-CV for TNF‐*α* = 9.67 ± 2.1%, IL‐1*β* = 5.17 ± 0.6%, and IL‐17A = 9.67 ± 2.1%; with sensitivity values of TNF‐*α* = 5.0 pg/mL, IL‐1*β* = 1.7 pg/mL, and IL‐17A = 4.0 pg/mL. The quantifications were made by interpolation from a standard curve for each cytokine studied by colorimetric measurements (450 nm) in an ELISA plate reader (Organon Teknika, Roseland, NJ, USA). The results are expressed in pg/mL.

### 2.11. Quantification of Proteins

Following the experimental procedures, 20 mg of lung tissue from each animal were removed from the thoracic cavity and transferred to Eppendorf tubes containing lysis buffer, Cell Lysis Buffer 5X (eBioscience, San Diego, CA), to form the homogenate.

The total protein dose was determined as previously reported [32]. Samples (5 *μ*L) from the lung tissue were added to 95 *μ*L distilled water plus 100 *μ*L Lowry reagent (25% copper-tartrate-carbonate, 25% sodium dodecyl sulfate 10%, 20% 0.8 N NaOH, 30% distilled water). After incubation at room temperature for 10 min, 50 *μ*L of Folin reagent (40% 1 N reactive Folin, 20% 2 N reactive Folin, and 40% distilled water) was added to the mixture and then incubated for 30 min. After this period, the mixture was pipetted into 96-well plates, and the total protein quantification was made by interpolation from a standard curve (0–40 *μ*g/*μ*L) by colorimetric measurements (630 nm) in an ELISA plate reader (Organon Teknika, Roseland, NJ, USA).

As suggested by the manufacturer, this procedure was performed to adjust the concentrations of each sample to 60 *μ*g protein/sample for determining protein phosphorylation.

### 2.12. Quantification of p65 NF-*κ*B Phosphorylation

For this protocol, a commercial kit containing monoclonal antibodies specific to phosphorylated mouse p65 protein (phospho-p65 NF-*κ*B (Ser536) Instant One ELISA kit, eBioscience, San Diego, California, USA) were used. The experimental protocol was performed according to the manufacturer's instructions. Colorimetric measurements (450 nm) were performed on an ELISA plate reader, and results are expressed as relative fold change compared with the negative control group, which represent the basal expression of phosphorylated p65 NF-*κ*B.

### 2.13. Quantification of p38 MAPK Phosphorylation

Samples of fluid leakage from mouse pleural cavities were adjusted using the Lowry method to contain the same protein concentrations (60 *μ*g). The samples were transferred to a plate with microwells containing specific monoclonal antibodies against the phosphorylated protein p38 MAPK (phospho-p38 MAPK (Tyr180/Tyr182) Instant One ELISA kit, eBioscience, San Diego, California, USA\). Colorimetric measurements (450 nm) were performed on an ELISA plate reader, and the results are expressed as relative fold change when compared with the negative control group; this was used to represent the basal expression of phosphorylated p38 MAPK.

### 2.14. Statistical Analysis

All data are expressed as mean ± standard error of the mean and percent of inhibition. The significant differences between groups were determined using analysis of variance followed by Newman–Keuls post hoc test. The results were analyzed using GraphPad Prism v5.0 Software (GraphPad Software Inc., San Diego, California, USA), and *P* values of <0.05 were considered significant.

## 3. Results

### 3.1. Phytochemical Analysis

**Figure 1:**
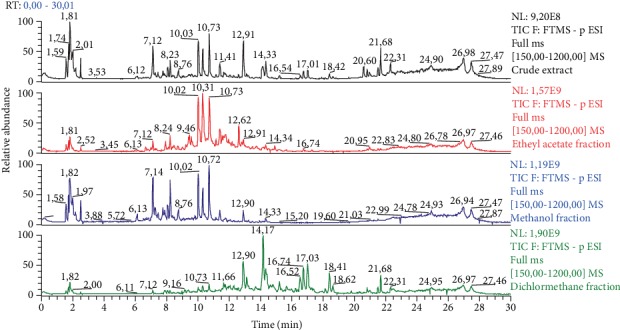
High-resolution electrospray ionization mass spectrometry (HRESIMS) total-ion chromatograms in the negative ion mode of crude extract and ethyl acetate, methanol and dichloromethane fractions. The compounds 3,4-diCQA (1), 3,5-diCQA (2), and 4,5-diCQA (3) possess retention time of 10.0, 10.31, and 10.73 min, respectively.

Analysis of the chemical composition of *C*. *pinnatifida* extract and organic fractions (DCM, EtOAc, and MeOH) using UHPLC–DAD–HRESIMS revealed the presence of several phenolic compounds (flavonoids, hydroxycinnamic acid derivatives, and chromenes). Based on the UV spectrum of the constituents with retention times 10.03, 10.31, and 10.73 min, the presence of chlorogenic acid derivatives was suggested (Figure 1). LC-MS^*n*^ analyses in the negative ion mode using an ESI orbitrap mass spectrometer and the hierarchical key proposed by Clifford and coworkers [33–36] for discriminating between isomers of dicaffeoylquinic acid enabled us to conclude that these chlorogenic acid derivatives were compounds (1) 3,4-diCQA, (2) 3,5-diCQA, and (3) 4,5-diCQA. The Hex fraction was not analyzed using UHPLC on reverse-phase column because nonpolar compounds may display high adsorption on this type of stationary phase.

Based on UHPLC analyses, the chromatographic separation of the EtOAc fraction led to the isolation and characterization of the three structural isomeric chlorogenic acid derivatives previously identified using UHPLC–DAD–HRESIMS: (1) 3,4-diCQA, (2) 3,5-diCQA, and (3) 4,5-diCQA. The compounds' structures were confirmed as described by Lima et al. [28]. Compound 2 was identified by comparing its 1D and 2D NMR spectroscopic data with those published in the literature by Lima et al. [37] and compounds 1 and 3 were distinguished based on their MS/MS fragmentation patterns in MS experiments and in the hierarchical fragmentation scheme previously established by Clifford and coworkers [33–36] to characterize chlorogenic acid derivatives.

### 3.2. Effect of CE, Fractions, and Isolated Compounds of C. pinnatifida on Relative Fold Change of Leukocytes and Exudate Protein Concentration

Doses of 50 and 100 mg/kg CE significantly inhibited leukocytes by 41.11 ± 3.76% and 41.69 ± 1.3%, respectively (*P* < 0.01). This inhibition was associated with a significant decrease in the relative fold change of neutrophils by 42.13 ± 0.99% and 42.13 ± 2.99%, respectively (*P* < 0.01). Moreover, at the same doses, CE inhibited exudate protein concentration by 20.25 ± 4.49% and 33.62 ± 4.76%, respectively (*P* < 0.05) (Table 1).

Doses of 10 and 25 mg/kg Hex fraction demonstrated a significant anti-inflammatory effect by decreasing the relative fold change of leukocytes by 48.19 ± 2.96% and 65.78 ± 1.38%, respectively (*P* < 0.01); this effect was due to the reduction on relative fold change of neutrophils by 51.03 ± 3.08% and 71.32 ± 1.09%, respectively (*P* < 0.01). Moreover, the Hex fraction decreased the exudate protein concentration by 26.66 ± 5.74% and 35.29 ± 7.94%, respectively (*P* < 0.01) (Table 1).

Doses of 5–25 mg/kg MeOH fraction reduced the relative fold change of leucocytes ranging from 42.34 ± 6.09% to 50.32 ± 2.98% (*P* < 0.01), and this inhibition was related to the decrease in relative fold change of neutrophils ranging from 42.31 ± 15.87% to 51.54 ± 4.61% (*P* < 0.01) (Table 1). However, only doses of 10 and 25 mg/kg of this fraction were effective in inhibiting the exudate protein concentration by 25.17 ± 6.46% and 29.23 ± 5.32%, respectively (*P* < 0.01) (Table 1).

Similar to the MeOH fraction, 5–25 mg/kg EtOAc fraction was effective in reducing the relative fold change of leukocytes ranging from 62.23 ± 3.42% to 66.23 ± 1.94% (*P* < 0.01), and this effect appeared to be related to the decrease in neutrophils ranging from 66.23 ± 4.12% to 71.23 ± 1.89% (*P* < 0.01). Moreover, the EtOAc fraction at three tested doses (5, 10, and 25 mg/kg) significantly decreased exudate protein concentration ranging from 33.49 ± 2.91% to 52.69 ± 8.83% (*P* < 0.01) (Table 1).

The 3,5- and 4,5-diCQA isomers inhibited leukocytes (% inhibition: 3,5-diCQA, 2.5 mg/kg: 27.78 ± 4.87% and 5 mg/kg: 37.08 ± 6.69%; 4,5-diCQA, 5 mg/kg: 41.40 ± 8.83%; *P* < 0.01). This inhibition profile was also due to the ability of these two compounds to reduce the relative fold change of neutrophils (% inhibition: 3,5-diCQA, 2.5 mg/kg: 26.23 ± 4.56% and 5 mg/kg: 41.99 ± 5.54%; 4,5-diCQA, 5 mg/kg: 43.55 ± 8.12%; *P* < 0.01). The two isomers also inhibited exudate protein concentration (% inhibition: 3,5-diCQA, 2.5 mg/kg: 31.79 ± 5.02% and 5 mg/kg: 36.96 ± 4.74%; 4,5-diCQA, 5 mg/kg: 54.89 ± 4.99%; *P* < 0.01) (Table 1).

The aqueous and DCM fractions and the isomer 3,4-diCQA did not inhibit leukocyte and exudate protein concentration (data not shown). In addition, CE, derived fractions, and isolated compounds did not inhibit mononuclear cells (data not shown).

The results revealed that CE (50 mg/kg), fractions (Hex, 10 mg/kg; MeOH, 10 mg/kg; and EtOAc, 5 mg/kg), and isolated compounds (3,5-diCQA, 2.5 mg/kg and 4,5-diCQA, 5 mg/kg) were effective in inhibiting leukocyte numbers and exudate protein concentration. These doses were used in the next experiments.

### 3.3. Effect of CE, Fractions, and Isolated Compounds from C. pinnatifida on MPO and ADA Activities and NO_*x*_ Levels

The MPO and ADA are important enzymes, which can be used as markers of activated neutrophils and mononuclear cells, respectively [38–40].

The CE, its derived fractions, and isolated compounds caused a significant decrease in MPO (% inhibition: CE, 37.73 ± 01.03%; Hex, 31.69 ± 2.16%; MeOH, 33.09 ± 2.38%; EtOAc, 30.25 ± 0.39%; 3,5-diCQA, 25.29 ± 2.39%; and 4,5-diCQA, 20.10 ± 2.93%; *P* < 0.01) (Table 2) and ADA levels (% inhibition: CE, 59.17 ± 10.16; Hex, 44.48 ± 6.72%; MeOH, 71.59 ± 5.78%; EtOAc, 81.05 ± 2.06; 3,5-diCQA, 70.06 ± 6.08%; and 4,5-diCQA, 47.87 ± 6.14%; *P* < 0.01) (Table 2).

Moreover, CE, its derived fractions, and isolated compounds significantly decreased NO_*x*_ levels (% inhibition: CE, 23.82 ± 14.67%; Hex, 40.37 ± 3.49%; MeOH, 26.68 ± 3.60%; EtOAc, 35.27 ± 03.11%; 3,5-diCQA, 33.60 ± 3.44%; and 4,5-diCQA, 29.63 ± 3.13%; *P* < 0.01) (Table 2).

### 3.4. Effect of CE, Fractions, and Isolated Compounds from *C*. *pinnatifida* on TNF-*α*, IL-1*β*, and IL-17A Levels

Only CE, EtOAc fraction, and isolated compounds significantly decreased TNF-*α* levels (% inhibition: CE, 54.50 ± 4.11%; EtOAc, 53.73 ± 3.03%; 3,5-diCQA, 70.15 ± 11.15%; and 4,5-diCQA, 56.45 ± 6.76%; *P* < 0.05) (Table 3).

The CE, fractions, and isolated compounds also decreased IL-1*β* levels (% inhibition: CE, 53.59 ± 1.15%; Hex, 57.20 ± 10.88%; MeOH, 50.29 ± 5.34%; EtOAc, 29.81 ± 4.29%; 3,5-diCQA, 79.94 ± 5.29%; and 4,5-diCQA, 76.33 ± 1.43%; *P* < 0.01) (Table 3) and IL-17A levels (% inhibition: CE, 55.87 ± 5.88%; Hex, 68.26 ± 23.11%; MeOH, 62.92 ± 12.81%; EtOAc, 42.99 ± 8.39%; 3.5-diCQA, 56.31 ± 12.73%; and 4.5-diCQA, 60.02 ± 18.50%; *P* < 0.05) (Table 3).

### 3.5. Effect of Isolated Compounds on the Phosphorylation of p65 NF-*κ*B and p38 MAPK

**Figure 2:**
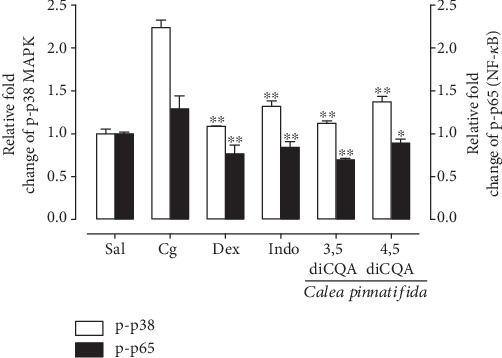
Effects of isolated compounds obtained from *Calea pinnatifida* leaves on p65 (p-p65 NF-*κ*B) and p38 (p-p38 MAPK) phosphorylation. 3,5-di-*O*-*E*-caffeoylquinic acid (3,5-diCQA: 2.5 mg/kg) and 4,5-di-*O*-*E*-caffeoylquinic acid (4,5-diCQA: 5 mg/kg) administered 0.5 h before pleurisy induction. Sal: negative control group, animals treated only with sterile saline; Cg: positive control group, animals treated only with carrageenan (1%); Dex: animals treated with dexamethasone (0.5 mg/kg) 0.5 h before pleurisy induction; Indo: animals treated with indomethacin (5 mg/kg) 0.5 h before pleurisy induction. Bars indicate the mean ± SEM of six animals. ^∗^*P* < 0.05 and ^∗∗^*P* < 0.01.

Our experiments showed that the isomers 3,5- and 4,5-diCQA were effective in inhibiting the phosphorylation of p65 NF-*κ*B by 46.23 ± 1.59% and 30.96 ± 3.48%, respectively (*P* < 0.05) and the phosphorylation of p38 MAPK by 50.00 ± 1.43% and 38.75 ± 2.83%, respectively (*P* < 0.01) (Figure 2).

## 4. Discussion

Our study demonstrated important anti-inflammatory activities of *C*. *pinnatifida.* This herb caused a significant decrease in leukocyte numbers and exudation. These effects appear to be related to the decrease in MPO and ADA activities and also production of mediators involved in the inflammatory response (NO and proinflammatory cytokines (TNF-*α*, IL-1*β*, and IL-17A)).

Our results are consistent with other studies for this genre, such as studies by Gómez and Gil [12] and Guevara et al. [41] who demonstrated that *C*. *prunifolia* inhibited both 12-*O*-tetradecanoylphorbol-13-acetate-induced ear edema and Cg-induced paw edema in mice. Studies have also demonstrated anti-inflammatory effects of the aqueous extract of *C*. *zacatechichi* that decreased neutrophil migration into the Cg-induced peritoneal inflammation in rats [42] and anti-inflammatory and antinociceptive effects of the MeOH extract of *C*. *zacatechichi* that inhibited Cg-induced paw edema and acetic acid-induced abdominal writhes in mice and rats, respectively [16]. Moreover, *in vitro* studies showed that the MeOH extract of *C*. *zacatechichi* also inhibited prostaglandin E2 production in activated macrophages induced by lipopolysaccharides (LPS) in the peritoneal cavity of rats [42].

The CE, derived fractions, and isolated compounds obtained from *C*. *pinnatifida* leaves inhibited leukocytes and exudate protein concentration. The EtOAc fraction showed greater inhibition of these inflammatory parameters because a lower dose of this fraction (5 mg/kg) decreased all the studied inflammatory parameters compared with that of CE and Hex and MeOH fractions, which inhibited the same inflammation at higher doses of 50, 25, and 10 mg/kg, respectively. Considering the isolated compounds, 3,5-diCQA showed a more potent anti-inflammatory effect than 4,5-diCQA because a lower dose of 3,5-diCQA (2.5 mg/kg) inhibited the inflammation parameters compared with that of 4,5 diCQA (5 mg/kg).

The inhibitory effect of neutrophils and MPO and ADA are correlated with the inhibition of activated cells because MPO is abundantly expressed in neutrophils and is directly related to the phagocytic activity of these cells [43]. Furthermore, ADA activity is important in the regulation of extracellular adenosine concentrations and consequently in stimulating receptors mainly involved in modulating the inflammatory response [44]. In pulmonary tissue and neutrophils, A_2A_ receptors mediate most of the anti-inflammatory effects of adenosine, and the expression of this receptor is increased in an inflamed lung [45–47].

The CE, derived fractions, and isolated compounds also inhibited the exudation process. This effect is associated with the ability of the plant material to decrease NO_*x*_ concentrations. NO has several roles in immune responses, including infection control, regulation of signaling cascades, transcription factors, rolling and migration of leukocytes, and cytokine production, and control of vascular responses [48]. *In vitro* studies corroborate with our results because the authors showed the inhibitory effect of 3,5-diCQA and 4,5-diCQA on NO production in LPS-induced RAW 264.7 cells, and this effect was related to the inhibition of inducible NO [25, 49].

The involvement of TNF-*α* and IL-1*β* in Cg-induced inflammation in mice has already been reported [50]. Cg activates toll-like receptor 4 and leads to the transcription of these cytokines [51]. TNF-*α* and IL-1*β* are important proinflammatory cytokines, mainly secreted by neutrophils and macrophages that play a central role in the onset and progression of the inflammatory responses [52]. The beneficial effects of TNF-*α* inhibition was demonstrated in the murine models of lung inflammation [53, 54]. Furthermore, studies showed that there is an increase in IL-1*β* concentrations in patients with COPD and neutrophilic asthma and also in the experimental models of smoke-induced COPD in mice [3, 55–57]. In our experiments, *C*. *pinnatifida* was effective in inhibiting TNF-*α* and IL-1*β*. Consistent with our findings, recent results demonstrated the ability of caffeoylquinic acids to inhibit TNF-*α* expression in LPS-stimulated RAW264.7 cells [24, 49] and decrease the release of IL-1*β* by TNF-*α*- stimulated human keratinocytes [58].

The CE, derived fractions, and compounds isolated from *C*. *pinnatifida* were also effective in decreasing IL-17A concentrations in pleural fluid after Cg-induced inflammation. IL-17A is another cytokine that has been gaining importance in the development of inflammatory disease [59]. This cytokine has multiple functions, such as stimulation of inflammatory cells, mediators' secretion, and neutrophil recruitment, which occur in synergism with TNF-*α* in the activation of endothelial cells [60]. The increased level of IL-17A or its mRNA were detected in sputum [61], bronchial tissues [62], and serum [63, 64] of patients with asthma. In addition, neutrophils in the airways of asthmatic patients appear to be related to the increased IL-17A expression [61].

It is well known that NF-*κ*B and MAPK pathways are involved in the inflammatory response. NF-*κ*B pathway increases the expression of many proinflammatory genes, including cytokines, chemokines, and adhesion molecules [65]. p38 MAPK plays a key role in the inflammatory responses via proinflammatory cytokine activation and production, NO synthase induction, cell proliferation, cell differentiation, and apoptosis induction [66].

To try understanding the anti-inflammatory mechanism of action of *C*. *pinnatifida*, we propose to analyzed the effect of the isolated compounds (3,5-diCQA and 4,5-diCQA) upon NF-*κ*B and MAPK pathways. Our results revealed that both isomers of caffeoylquinic acid inhibited the phosphorylation of p65 NF-*κ*B and p38 MAPK. These findings are consistent with those of our previous study, which showed the inhibitory effect of a mixture of diCQA isomers on p65 NF-*κ*B phosphorylation [27]. Furthermore, recent studies have shown that 3,5-diCQA and 4,5-diCQA inhibit p65 nuclear translocation in LPS-induced RAW 264.7 macrophages by preventing the degradation of I*κ*B*α*, an important inhibitory protein in the p65 NF-*κ*B pathway [24, 25]. Similar to our findings, a study assessing the effect of a polyphenol mixture isolated from *Lonicera japonica* Thunb. containing diCQAs showed the ability of diCQAs to inhibit the phosphorylation of p38 MAPK in LPS-stimulated RAW 246.7 macrophages [67]. Furthermore, inhibition of this pathway by diCQAs present in a propolis MeOH extract was observed in another *in vitro* study using LPS-stimulated RAW 246.7 macrophages, and these compounds were effective in inhibiting p38 MAPK phosphorylation [68].

## 5. Conclusion

In summary, we demonstrate for the first time the anti-inflammatory effects of *C*. *pinnatifida* in an *in vivo* model of lung inflammation. The CE, derived fractions, and isolated compounds showed significant anti-inflammatory properties by inhibiting leukocytes and exudation and decreasing the activities of ADA and MPO and the concentrations of important proinflammatory mediators, such as NO, TNF-*α*, IL-1*β*, and IL-17A. These effects are related to the ability of 3,5-diCQA and 4,5-diCQA to act on p65 NF-*κ*B and p38 MAPK phosphorylation, two important intracellular pathways in the progression and maintenance of inflammatory processes.

## Figures and Tables

**Table 1:** Effects of CE, fractions, and isolated compounds obtained from *C*. *pinnatifida* leaves upon relative fold change of leukocytes and exudate protein concentration in a murine model of carrageenan-induced pleurisy.

Groups (mg/kg)	Relative fold change of leukocytes	Relative fold change of neutrophils	Exudate protein concentration (*μ*g/mL)
Sal^a^	—	—	2.34 ± 0.15
Cg^a^	5.11 ± 0.31	8.63 ± 0.12	16.00 ± 0.85
CE (25)^b^	4.88 ± 0.07	8.09 ± 0.20	14.45 ± 1.01
CE (50)^b^	3.01±0.0^∗∗^	4.85±0.17^∗∗^	12.75 ± 0.71^∗^
CE (100)^b^	2.98±0.03^∗∗^	4.86±0.08^∗∗^	10.61±0.76^∗∗^
Hex (5)^b^	4.88 ± 0.09	8.09 ± 0.22	15.20 ± 1.85
Hex (10)^b^	1.64±0.10^∗∗^	2.37±0.13^∗∗^	11.73±0.91^∗∗^
Hex (25)^b^	2.56±0.19^∗∗^	4.13±0.26^∗∗^	10.35±1.17^∗∗^
MeOH (5)^b^	2.83±0.93^∗∗^	4.82±1.01^∗∗^	20.18 ± 0.17
MeOH (10)^b^	2.46±0.19^∗∗^	4.13±0.21^∗∗^	11.32±0.85^∗∗^
MeOH (25)^b^	2.69±0.27^∗∗^	4.58±0.29^∗∗^	11.97±1.03^∗∗^
EtOAc (2.5)^b^	4.79 ± 0.16	8.16 ± 0.19	15.72 ± 0.65
EtOAc (5)^b^	1.85±0.08^∗∗^	2.82±0.31^∗∗^	8.83±0.35^∗∗^
EtOAc (10)^b^	1.77±0.09^∗∗^	2.62±0.13^∗∗^	7.56±1.41^∗∗^
EtOAc (25)^b^	1.64±0.15^∗∗^	2.36±0.12^∗∗^	10.64±0.46^∗∗^
3,5-diCQA (1)^b^	5.23 ± 0.16	8.47 ± 0.09	15.05 ± 0.47
3,5-diCQA (2.5)^b^	3.69±0.21^∗∗^	6.14±0.19^∗∗^	10.91±0.80^∗∗^
3,5-diCQA (5)^b^	3.21±0.22^∗∗^	4.91±0.26^∗∗^	10.08±0.75^∗∗^
4,5-diCQA (1)^b^	4.74 ± 0.20	8.09 ± 0.12	15.15 ± 0.63
4,5-diCQA (2.5)^b^	5.22 ± 0.31	8.82 ± 0.31	14.36 ± 0.75
4,5-diCQA (5)^b^	3.00±0.19^∗∗^	4.83±0.47^∗∗^	7.21±0.80^∗∗^
Dex (0.5)^b^	2.49±0.21^∗∗^	4.22±0.23^∗∗^	10.15±0.45^∗∗^
Indo (5)^b^	2.83±0.27^∗∗^	4.75±0.36^∗∗^	11.41±0.50^∗∗^

Crude extract (CE: 25–100 mg/kg), hexane fraction (Hex: 5–25 mg/kg), methanol fraction (MeOH: 5–25 mg/kg), ethyl acetate fraction (EtOAc: 2.5–25 mg/kg), 3,5-di-*O*-*E*-caffeoylquinic acid (3,5-diCQA: 1–5 mg/kg), and 4,5-di-*O*-*E*-caffeoylquinic acid (4,5-diCQA: 1–5 mg/kg) obtained from *C*. *pinnatifida* leaves administered 0.5 h before pleurisy induction. Sal: negative control group, animals treated only with sterile saline (0.95%); Cg: positive control group, animals treated only with carrageenan (1%); Dex: animals treated with dexamethasone (0.5 mg/kg) 0.5 h before pleurisy induction; Indo: animals treated with indomethacin (5 mg/kg) 0.5 h before pleurisy induction. ^a^Administered by intrapleural route (i.pl.). ^b^Administered by intraperitoneal route (i.p.). Each group represents the mean ± SEM of six animals. ^∗^*P* < 0.05 and ^∗∗^*P* < 0.01.

**Table 2:** Effects of CE, fractions, and isolated compounds obtained from *C*. *pinnatifida* leaves upon myeloperoxidase (MPO) and adenosine deaminase (ADA) activities and nitrate/nitrite (NO_*x*_) levels in a murine model of carrageenan-induced pleurisy.

Groups (mg/kg)	MPO (mU/mL)	ADA (U/L)	NO_*x*_ (*μ*M)
Sal^a^	64.47 ± 1.63	3.09 ± 0.25	8.48 ± 0.22
Cg^a^	114.2 ± 16.54	21.82 ± 2.00	18.36 ± 1.34
CE (50)^b^	71.09±1.81^∗∗^	8.91±2.22^∗∗^	13.98 ± 2.69^∗^
Hex (25)^b^	77.99±2.47^∗∗^	12.12±1.47^∗∗^	10.94±0.64^∗∗^
MeOH (10)^b^	76.39±2.72^∗∗^	6.20±1.26^∗∗^	13.45 ± 0.66^∗^
EtOAc (5)^b^	79.63±0.46^∗∗^	4.14±0.45^∗∗^	11.88±0.57^∗∗^
3,5-diCQA (2.5)^b^	85.30±2.74^∗∗^	6.53±1.33^∗∗^	12.18±0.63^∗∗^
4,5-diCQA (5)^b^	91.23±3.35^∗∗^	11.38±1.34^∗∗^	12.91±0.58^∗∗^
Dex (0.5)^b^	64.98±2.98^∗∗^	10.18±0.75^∗∗^	9.69±0.55^∗∗^
Indo (5)^b^	67.44±4.81^∗∗^	10.49±0.99^∗∗^	11.54±0.40^∗∗^

Crude extract (CE: 50 mg/kg), hexane fraction (Hex: 25 mg/kg), methanol fraction (MeOH: 10 mg/kg), ethyl acetate fraction (EtOAc: 5 mg/kg), 3,5-di-*O*-*E*-caffeoylquinic acid (3,5-diCQA: 2.5 mg/kg), and 4,5-di-*O*-*E*-caffeoylquinic acid (4,5-diCQA: 5 mg/kg) obtained from *C. pinnatifida* leaves administered 0.5 h before pleurisy induction. Sal: negative control group, animals treated only with sterile saline (0.95%); Cg: positive control group, animals treated only with carrageenan (1%); Dex: animals treated with dexamethasone (0.5 mg/kg) 0.5 h before pleurisy induction; Indo: animals treated with indomethacin (5 mg/kg) 0.5 h before pleurisy induction. ^a^Administered by intrapleural route (i.pl.). ^b^Administered by intraperitoneal route (i.p.). Each group represents the mean ± SEM of six animals. ^∗^*P* < 0.05 and ^∗∗^*P* < 0.01.

**Table 3:** Effects of CE, fractions, and isolated compounds of *C*. *pinnatifida* upon tumor necrosis factor-alpha (TNF-*α*), interleukin-1*β* (IL-1*β*), and interleukin-17A (IL-17A) in a murine model of carrageenan-induced pleurisy.

Groups (mg/kg)	TNF-*α* (pg/mL)	IL-1*β* (pg/mL)	IL-17A (pg/mL)
Sal^a^	85.05 ± 8.35	115.1 ± 21.72	98.90 ± 5.62
Cg^a^	991.3 ± 2.27	1545.00 ± 170.50	337.00 ± 1.14
CE (50)^b^	451.10±40.76^∗∗^	717.10±17.78^∗∗^	148.70±19.85^∗∗^
Hex (25)^b^	895.50 ± 50.11	661.30±168.10^∗∗^	124.40±69.29^∗∗^
MeOH (10)^b^	809.00 ± 110.10	768.00±82.63^∗∗^	125.00±43.16^∗∗^
EtOAc (5)^b^	458.60±30.16^∗∗^	1085.00±66.39^∗∗^	192.10 ± 28.31^∗^
3,5-diCQA (2.5)^b^	296.00±110.60^∗∗^	310.00±81.82^∗∗^	147.20±42.91^∗∗^
4,5-diCQA (5)^b^	431.7±67.08^∗∗^	365.80±22.10^∗∗^	172.40±34.85^∗∗^
Dex (0.5)^b^	371.00±58.24^∗∗^	243.3±6.00^∗∗^	143.80±42.47^∗∗^
Indo (5)^b^	415.80±64.96^∗∗^	467.8±117.50^∗∗^	124.10±40.46^∗∗^

Crude extract (CE: 50 mg/kg), hexane fraction (Hex: 25 mg/kg), methanol fraction (MeOH: 10 mg/kg), ethyl acetate fraction (EtOAc: 5 mg/kg), 3,5-di-*O*-*E*-caffeoylquinic acid (3,5-diCQA: 2.5 mg/kg), and 4,5-di-*O*-*E*-caffeoylquinic acid (4,5-diCQA: 5 mg/kg) obtained from *C*. *pinnatifida* leaves administered 0.5 h before pleurisy induction. Sal: negative control group, animals treated only with sterile saline (0.95%); Cg: positive control group, animals treated only with carrageenan (1%); Dex: animals treated with dexamethasone (0.5 mg/kg) 0.5 h before pleurisy induction; Indo: animals treated with indomethacin (5 mg/kg) 0.5 h before pleurisy induction. ^a^Administered by intra-pleural route (i.pl.). ^b^Administered by intra-peritoneal route (i.p.). Each group represents the mean ± SEM of six animals. ^∗^*P* < 0.05 and ^∗∗^*P* < 0.01.

## Data Availability

No data were used to support this study.

## Abbreviations

ADA: Adenosine deaminase
CE: Crude extract
Cg: Carrageenan
DCM: Dichloromethane
Dex: Dexamethasone
EtOAc: Ethyl acetate
Indo: Indomethacin
IL-1*β*: Interleukin-1-beta
IL-17A: Interleukin-17A
ELISA: Enzyme-linked immunosorbent assay
Hex: Hexane
MAPK: Mitogen-activated protein kinases
MeOH: Methanol
MPO: Myeloperoxidase
NF-*κ*B: Nuclear transcription factor kappa B
NO: Nitric oxide
NOx: Nitric oxide metabolites
TNF-*α*: Tumor necrosis factor-alpha.
